# Hemorrhagic Pneumonia Caused by *Stenotrophomonas maltophilia* in Patients with Hematologic Malignancies—A Systematic Review and Meta-Analysis

**DOI:** 10.3390/medicina60010162

**Published:** 2024-01-15

**Authors:** Chienhsiu Huang, Sufang Kuo, Lichen Lin

**Affiliations:** 1 Department of Internal Medicine, Dalin Tzu Chi Hospital, Buddhist Tzu Chi Medical Foundation, No. 2, Min-Sheng Road, Dalin Town, Taiwan; 2 Department of Nursing, Dalin Tzu Chi Hospital, No. 2, Min-Sheng Road, Dalin Town, Taiwan; dl08596@tzuchi.com.tw (S.K.); df462594@tzuchi.com.tw (L.L.)

**Keywords:** *Stenotrophomonas maltophilia*, hematologic malignancy, hemorrhagic pneumonia, neutropenia, thrombocytopenia

## Abstract

*Background and Objectives:* There is a need for information regarding the clinical picture of hemorrhagic pneumonia caused by *Stenotrophomonas maltophilia* in patients with hematologic malignancies. In this study, we aimed to investigate the risk factors associated with hemorrhagic pneumonia caused by *Stenotrophomonas maltophilia. Materials and Methods*: A review of the clinical picture of hemorrhagic pneumonia based on reported cases in the literature was performed. In addition, patients with hematologic malignancies who had a *Stenotrophomonas maltophilia* infection were included in the meta-analysis to evaluate risk factors for hemorrhagic pneumonia. *Results*: A total of 91 patients had hemorrhagic pneumonia. Acute myeloid leukemia was present in 57 patients (62.6%). Those with bacteremia accounted for 94%, while those with neutropenia accounted for 95% and those with thrombocytopenia accounted for 86.7%. Hemorrhagic pneumonia was a risk factor for mortality of *Stenotrophomonas maltophilia* infection in patients with hematologic malignancies. Neutropenia and thrombocytopenia were identified as risk factors for hemorrhagic pneumonia. *Conclusions*: *Stenotrophomonas maltophilia* bacteremia with hemorrhagic pneumonia in patients with hematologic malignancies is a situation with rapid development and high mortality. Neutropenia and thrombocytopenia were risk factors for hemorrhagic pneumonia in patients with hematologic malignancies and with *Stenotrophomonas maltophilia* bacteremia; thus, these patients should be managed with caution.

## 1. Introduction

*Stenotrophomonas maltophilia* is a gram-negative bacterium. After being identified in 1943 as Bacterium bookeri, *Stenotrophomonas maltophilia* was classified as the genus Pseudomonas in 1961. In 1983, it was placed in the genus Xanthomonas, and then in 1993 it was permanently assigned to the genus Stenotrophomonas [1].

*Stenotrophomonas maltophilia* has been identified as a novel nosocomial pathogen associated with life-threatening invasive infections and a high mortality rate in some patient populations, especially those who are severely ill or immunocompromised [2,3,4]. Numerous intrinsic and acquired resistance traits make *Stenotrophomonas maltophilia*-caused infections notoriously difficult to treat. *Stenotrophomonas maltophilia* shows significant levels of inherent resistance to a range of antibiotics with different structures, such as tetracycline, quinolones, aminoglycosides, and beta-lactams [5,6]. Furthermore, it employs a wide spectrum of resistance mechanisms. Reduced permeability, the development of multidrug efflux pumps, and the manufacture of beta-lactamase, carbapenemase, and aminoglycoside-modifying enzymes are some of the resistance mechanisms used by *Stenotrophomonas maltophilia* [7,8]. Thus, choosing the optimal antibiotic for the treatment of *Stenotrophomonas maltophilia* bacteremia is notoriously challenging. Long-term hospitalization necessitating invasive procedures, prior use of broad-spectrum antibiotics, the need for mechanical ventilation, and patients with hematologic malignancies are risk factors for infection by *Stenotrophomonas maltophilia* [9,10]. Mortality rates from *Stenotrophomonas maltophilia* infections are high, ranging from 21% to 69%, according to a study of the literature [11].

*Stenotrophomonas maltophilia* infection has recently become a significant issue in the treatment of hematologic malignancy patients. It can result in severe infections such as bacteremia or hemorrhagic pneumonia [12,13,14]. Patients with hematologic malignancies are at very high risk for *Stenotrophomonas maltophilia* blood stream infections due to prolonged healthcare exposures, high rates of broad-spectrum antibiotic usage, and impaired immune systems [15,16,17]. According to the study of Cho, S.Y. et al., patients with hematologic malignancies had an overall mortality rate of *Stenotrophomonas maltophilia* blood stream infection of 64.5% and an attributable mortality rate of 38.7%. Shock at the commencement of blood stream infection, severe neutropenia, and pneumonia were independent risk factors for mortality [18]. In patients with hematologic malignancies who had *Stenotrophomonas maltophilia* bacteremia, the total 14-day mortality rate was 54.2%, and the 30-day mortality rate was 61.0%, showing that *Stenotrophomonas maltophilia* bacteremia caused much greater mortality than other gram-negative bacillin [19]. More crucially, *Stenotrophomonas maltophilia* infection results in fulminant and deadly hemorrhagic pneumonia in patients with hematologic malignancies who have severe and persistent neutropenia. The majority of patients pass away within a few days of hemoptysis starting and before the isolate is found in blood or sputum cultures [3].

The importance of clinical guidance is limited because very few cases of hemorrhagic pneumonia caused by *Stenotrophomonas maltophilia* in patients with hematologic malignancies have been reported in the literature. The risk factors for hemorrhagic pneumonia in patients with hematologic malignancies are unclear. There has been no systematic evaluation of the risk factors for hemorrhagic pneumonia in patients with hematologic malignancies. The purpose of this review is to inform the development of preventative and therapeutic options for hemorrhagic pneumonia in patients with hematologic malignancies in the hope of reducing the mortality rates associated with this infection.

## 2. Methods

### 2.1. Systematic Review

This was a systematic review of hemorrhagic pneumonia based on previously reported cases in the literature. We searched PubMed and WOS for all pertinent information regarding *Stenotrophomonas maltophilia* infection up to 31 May 2023, and identified all hematologic malignancy patients with *Stenotrophomonas maltophilia*-caused hemorrhagic pneumonia.

### 2.2. Definitions

*Stenotrophomonas maltophilia* bacteremia was defined as the isolation of *Stenotrophomonas maltophilia* from a single blood culture. When signs or symptoms of systemic infection co-occurred with bacteremia, a case was considered clinically significant. Pneumonia caused by this organism was characterized when *Stenotrophomonas maltophilia* was isolated from the blood or respiratory specimens. Cough with or without sputum production, chest pain, dyspnea, and changed breathing sounds on auscultation were also needed, and pneumonia was also seen on radiographs. Patients with hemorrhagic pneumonia were those with endotracheal tube hemorrhage, hemoptysis, or persistent blood in their sputum. Patients with a neutrophil count in peripheral blood below 500/mm^3^ were defined as neutropenic. Patients with peripheral blood platelet counts below 50,000/mm^3^ were defined as thrombocytopenia.

### 2.3. Meta-Analysis

The literature search was performed using PubMed, Web of Science, and Cochrane.

Library databases were used to identify all included clinical studies and meta-analyses or systematic reviews on the topic from 1 January 1990 to 31 May 2023. In the databases, we used the following search string: (*Stenotrophomonas maltophilia*) (bacteremia) (hemorrhagic pneumonia) (hematologic malignancy) (neutropenia) and (thrombocytopenia). Previously published systematic reviews and meta-analyses were reviewed to identify any additional studies that may have been missed in the primary literature search. Only English articles were included.

### 2.4. Inclusion Criteria

All studies included patients with hematologic malignancies who had *Stenotrophomonas maltophilia* bacteremia, including those who had hemorrhagic pneumonia and those who did not. If they reported one or more elements of the clinical information, all studies were included in the meta-analysis. The clinical information included the total number of patients who died, neutropenia patients, and thrombocytopenia patients.

### 2.5. Statistical Analysis

The RevMan 5, the Cochrane Review Manager software (RevMan, New York, NY, USA), was used to conduct statistical analyses. Fixed-effects and random-effects were utilized for data analysis. Statistical heterogeneity was evaluated using the Q-test and the I2 statistical techniques. Significant heterogeneity between the studies was defined as an I2 more than 50% and a *p* value for the Q-test less than 0.10 for each study. We tabulate the study intervention features and compare them to the scheduled groups for each synthesis using the forest plots. The funnel plot was examined to determine the degree of publication bias.

## 2.6. Institutional Review Board Statement

This review was performed in accordance with the PRISMA (Preferred Reporting Items for Systematic Reviews and Meta-Analyses) guidelines. The Systematic Review and Meta-Analysis was registered at the Prospero international prospective register of systematic reviews (registration: CRD42023461215).

## 3. Results

### 3.1. Characteristics of the Included Trials

**Figure 1:**
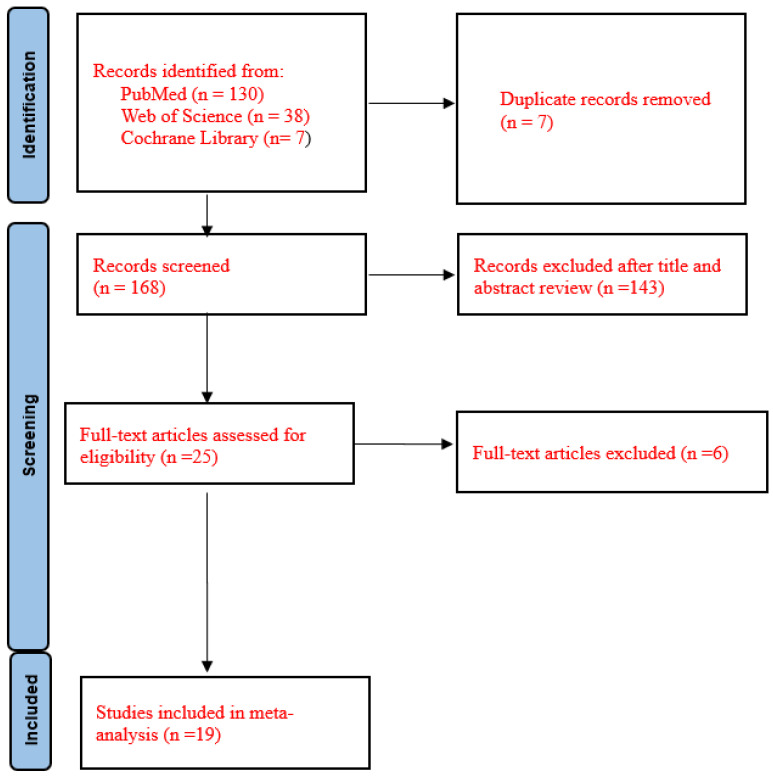
Flow diagram of the study selection process.

The details of the study selection process are shown in Figure 1. The numbers of studies from the initial search results from PubMed, Web of Science, and the Cochrane Library were 130, 38, and 7, respectively. There were seven duplicate articles. A total of 143 irrelevant studies were identified by reading the title and/or abstract. After excluding duplicates and irrelevant studies, 25 potentially relevant articles remained. After full-text article review, six articles were excluded because there were no reported hemorrhagic pneumonia cases caused by Stenotrophomonas maltophilia.

### 3.2. Patient Characteristics

In the literature, we discovered a total of 93 instances of hemorrhagic pneumonia caused by *Stenotrophomonas maltophilia*, including two cases involving newborns [20] and 91 cases with hematologic malignancies (Table 1) [21,22,23,24,25,26,27,28,29,30,31,32,33,34,35,36,37,38,39]. Table 2 provides a summary of the extensive clinical data for the 91 patients. There were 54 males and 37 females. The mean age was 45.19 years. Acute myeloid leukemia was present in 58 patients (63.7%), acute lymphoblastic leukemia in 15, myelodysplastic syndrome in 5, chronic myelogenous leukemia in 2, non-Hodgkin’s lymphoma in 8, hemophagocytic syndrome in 1, aggressive NK/T-cell leukemia in 1, and myelofibrosis in 1. Those with bacteremia accounted for 94%, while those with neutropenia accounted for 95%. A total of 86.7% (59/68) of the patients had thrombocytopenia because the platelet counts were not recorded for 23 of the patients.

### 3.3. Outcome

Hemorrhagic pneumonia was responsible for the deaths of 85.9% (55/64) of patients within 7 days and 90.1% (82/91) of patients within 30 days. Even after receiving prompt antibiotic treatment, only nine patients with hemorrhagic pneumonia survived.

### 3.4. Meta-Analysis of Stenotrophomonas maltophilia Bacteremia in Patients with Hematologic Malignancies

Patients with hematologic malignancies who have *Stenotrophomonas maltophilia* bacteremia have been described in five studies, including those who have hemorrhagic pneumonia and those who do not [21,28,29,32,38]. All five studies had a significant risk of bias and were retrospective in nature.

**Figure 2:**
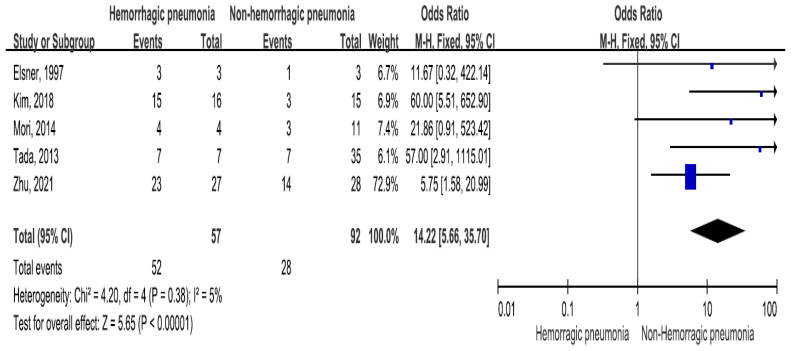
The *Stenotrophomonas maltophilia* bacteremia-related mortality between patients with hemorrhagic pneumonia and patients without hemorrhagic pneumonia [21,28,29,32,38].

The mortality rate of *Stenotrophomonas maltophilia* bacteremia was reported in five trials, which included 57 patients with hemorrhagic pneumonia (mortality rate 91.2%) and 92 patients without hemorrhagic pneumonia (mortality rate 30.4%) [21,28,29,32,38]. *Stenotrophomonas maltophilia* bacteremia-related mortality significantly differed between the two groups (OR = 14.22, 95% CI = 5.66–35.70, *p* < 0.001, I2 = 5%) (Figure 2).

**Figure 3:**
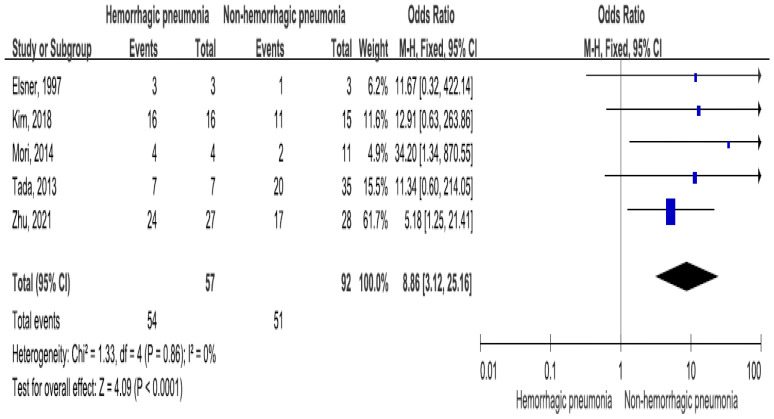
The neutropenia between patients with hemorrhagic pneumonia and patients without hemorrhagic pneumonia [21,28,29,32,38].

Five studies reported neutropenia of *Stenotrophomonas maltophilia* bacteremia, including 57 hemorrhagic pneumonia patients (94.7% patients with neutropenia) and 92 patients without hemorrhagic pneumonia (55.4% patients with neutropenia) [21,28,29,32,38]. There was a significant difference in *Stenotrophomonas maltophilia* bacteremia-related neutropenia between the two groups (OR = 8.86, 95% CI = 3.12–25.16, *p* < 0.001, I2 = 0%) (Figure 3).

**Figure 4:**
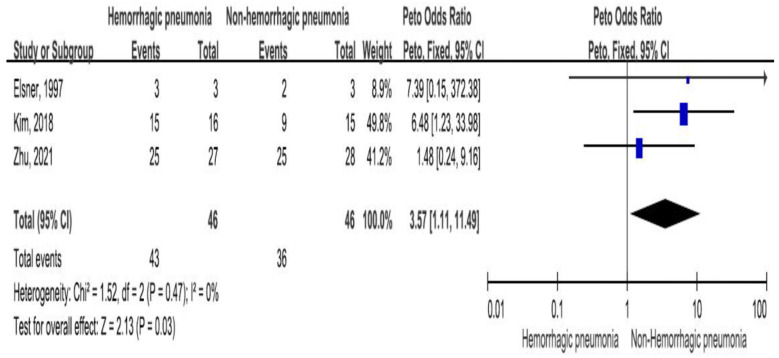
The thrombocytopenia between patients with hemorrhagic pneumonia and patients without hemorrhagic pneumonia [21,32,38].

Three studies reported thrombocytopenia of *Stenotrophomonas maltophilia* bacteremia, including 46 patients with hemorrhagic pneumonia (93.5% patients with thrombocytopenia) and 46 patients without hemorrhagic pneumonia (78.2% patients with thrombocytopenia) [21,32,38]. There was a significant difference in *Stenotrophomonas maltophilia* bacteremia-related thrombocytopenia between the two groups (OR = 3.57, 95% CI = 1.11–11.49, *p* = 0.03, I2 = 0%) (Figure 4).

### 3.5. Survival Cases

Nine survival cases have been reported in the literature. A 61-year-old female acute lymphoblastic leukemia patient with alveolar hemorrhage, acute respiratory distress syndrome, and *Stenotrophomonas maltophilia* isolation from sputum culture was presented by Andrei, S. et al. After receiving therapy with colistin and trimethoprim-sulfamethoxazole (TMP-SMX) and seeing a recovery of pneumonia, the patient had a positive outcome and was released from critical care after 26 days [35]. Penagos, S.C. et al. reported that the early use of combination therapy with TMP/SMX, polymyxin, and/or moxifloxacin resulted in good treatment results for two hematologic malignancy patients with hemorrhagic pneumonia caused by *Stenotrophomonas maltophilia*. Management-related factors might be to blame for the results of these two cases differing from others described in the literature. The use of TMP/SMX in different dosages and combinations as well as the timely initiation of appropriate treatment are examples of management variations. Regarding the quick commencement of appropriate care, in each of the described instances, at least one active antibiotic against *Stenotrophomonas maltophilia* (TMP/SMX) was begun within 48 h. First-line TMP/SMX and polymyxin-based treatment was given to patients. This combination has demonstrated in vitro synergism against *Stenotrophomonas maltophilia*, possibly as a result of TMP/SMX’s enhanced entrance following polymyxin disruption of the outer membrane’s permeability [34].

A 46-year-old man who had *Stenotrophomonas maltophilia* hemorrhagic pneumonia after consolidation chemotherapy for acute myeloid leukemia was the subject of a case study by Saito, K. et al. His respiratory condition was stabilized by ECMO, which gave TMP/SMX to treat the pneumonia. Because ECMO is primarily used to stabilize respiratory status, pneumonia with accessible evidence-based antimicrobial therapy and a possible curable hematological malignancy are necessary [37]. According to past studies, patients with persistent respiratory failure who are 65 years of age or older should not utilize ECMO. Due to the significant physiological demands of chemotherapy on elderly patients, age is a significant prognostic factor in hematological malignancies [40,41]. There were four survival cases in the Zhu series and four cases treated with early combination therapy with TMP/SMX, cefoperazone/sulbactam, moxifloxacin, and/or tigecycline and rapid recovery of neutropenia [38]. In addition, there is one survival case in the Kim SH series, and the author did not report in detail the clinical course of the successfully treated cases, nor did they make any comments on this issue [32].

## 4. Discussion

There were two major takeaways from this study. First, *Stenotrophomonas maltophilia* bacteremia in patients with hematologic malignancies who also had hemorrhagic pneumonia had a much greater mortality rate than those who did not. Hemorrhagic pneumonia was a risk factor for mortality. Second, in patients with hematologic malignancies with *Stenotrophomonas maltophilia* bacteremia, neutropenia and thrombocytopenia were identified as risk factors for hemorrhagic pneumonia.

### 4.1. Clinical Characteristic of Stenotrophomonas maltophilia Bacteremia with Hemorrhagic Pneumonia in Patients with Hematologic Malignancies

A review (52 cases of hemorrhagic pneumonia in patients with hematologic malignancies) by Kim, S.H. et al. showed that patients in the general population with *Stenotrophomonas maltophilia* bacteremia rarely experienced hemorrhagic pneumonia. Hemorrhagic pneumonia is a distinct clinical manifestation frequently observed in patients with hematologic malignancies. Patients with hemorrhagic pneumonia were eight times more likely to develop acute leukemia than patients with non-Hodgkin lymphoma. Neutropenia and thrombocytopenia were common clinical findings in patients with hemorrhagic pneumonia [32].

In the present study, we gathered data from 91 patients with hematologic malignancies who had hemorrhagic pneumonia. The most common underlying hematologic malignancy was acute leukemia. Of the 72 patients with acute leukemia, 57 (62.6%) were diagnosed with acute myeloid leukemia, and 15 (16.5%) were diagnosed with acute lymphoblastic leukemia. In our series, neutropenia (95%) and thrombocytopenia (86.7%) were also frequently observed. Within 7 days, hemorrhagic pneumonia was the cause of mortality for 85.9% (55/64) of patients, and within 30 days, it was the cause of mortality for 90.1% (82/91) of patients. In the current study, we offered the most in-depth and comprehensive clinical information on hemorrhagic pneumonia in patients with hematologic malignancies.

### 4.2. Mortality of Stenotrophomonas maltophilia Bacteremia with Hemorrhagic Pneumonia in Hematologic Malignancy Patients

Patients with hematologic malignancies are disproportionately likely to die from *Stenotrophomonas maltophilia* bacteremia. Bao, H. et al. showed that overall, 44.1% of patients with *Stenotrophomonas maltophilia* bacteremia and with hematologic malignancies died within 30 days, and inadequate initial antimicrobial therapy was a significant risk factor for a poor outcome [10]. Hematologic malignancies place patients at risk for *Stenotrophomonas maltophilia* infection because of their high exposure to broad-spectrum antibiotics and prolonged use of chemotherapy. More crucially, *Stenotrophomonas maltophilia* infection results in fulminant and fatal hemorrhagic pneumonia in patients with hematologic malignancies who have severe and persistent neutropenia. The most serious manifestation of *Stenotrophomonas maltophilia* bacteremia in patients with hematological abnormalities is hemorrhagic pneumonia. In the study of Kim, S.H. et al., 118 people with hematologic malignancies died overall in the first 30 days at a rate of 61.0%. Hemorrhagic pneumonia was found to be the most important risk factor for mortality. The majority of patients pass away within a few days of hemoptysis starting [32]. Zhu, L. et al. showed that patients with hematologic malignancies who develop *Stenotrophomonas maltophilia* bacteremia with hemorrhagic pneumonia have a very rapid progression and poor prognosis. In comparison to patients without hemorrhagic pneumonia, the mortality rate for *Stenotrophomonas maltophilia* bacteremia was much greater. In hematologic malignancy patients with *Stenotrophomonas maltophilia* bacteremia, hemorrhagic pneumonia was a significant risk factor for 30-day mortality. Nevertheless, the rate of receiving adequate initial antimicrobial therapy was similar between the hemorrhagic pneumonia group and the nonhemorrhagic pneumonia group. This suggests that despite effective initial antimicrobial therapy, hemorrhagic pneumonia caused by *Stenotrophomonas maltophilia* is a serious illness that makes it difficult to save a patient once it develops [38]. The Infectious Diseases Society of America treatment recommendations for moderate to severe *Stenotrophomonas maltophilia* infections include three methods: (1) the utilization of preferred combination treatment, namely, TMP-SMX and minocycline; (2) if there is a delay in clinical improvement with TMP-SMX monotherapy, a second drug (minocycline [recommended], tigecycline, levofloxacin, or cefiderocol) might be added; or (3) ceftazidime/eavibactam and aztreonam in combination when other agents are expected to be inactive or intolerable [42].

*Stenotrophomonas maltophilia* bacteremia has a significant mortality rate and is not well treated with the antibiotics often given to patients with hematologic malignancies. Since *Stenotrophomonas maltophilia*-specific antibiotics are seldom used to treat febrile neutropenia, it is critical to understand what variables contribute to patient mortality in hematologic malignancy patients with *Stenotrophomonas maltophilia* bacteremia. Due to the clinically severe state and significant mortality, patients with *Stenotrophomonas maltophilia* bacteremia and hemorrhagic pneumonia should receive early treatment with TMP-SMX base combination therapy. Four patients received combination therapy with TMP/SMX, cefoperazone/sulbactam, moxifloxacin, and/or tigecycline in Zhu’s series [38]. Penagos’s trial reported on two patients who were effectively treated following early combination treatment with TMP/SMX, polymyxin, and/or moxifloxacin [34]. Additionally, Andrei, S. et al. reported that hemorrhagic pneumonia resolved after treatment with colistin and TMP/SMX [35]. From the experience of the aforementioned seven successful instances, the preferred second medications may include polymyxin, colistin, and moxifloxacin. However, there has been no conclusive research into the effectiveness of combination treatment regimens for *Stenotrophomonas maltophilia* hemorrhagic pneumonia. Further clinical trials and clinical experiences by medical professionals are necessary to establish the effectiveness of combination therapy for *Stenotrophomonas maltophilia* bacteremia with hemorrhagic pneumonia.

### 4.3. Neutropenia Is a Risk Factor for Hemorrhagic Pneumonia in Stenotrophomonas maltophilia Bacteremia of Hematological Malignancy Patients

Regarding neutropenia, the current study found a significant difference in the percentage of neutropenia between hemorrhagic pneumonia patients and nonhemorrhagic pneumonia patients. Therefore, preventing *Stenotrophomonas maltophilia* infection and the subsequent development of hemorrhagic pneumonia is of paramount importance. Hemorrhagic pneumonia risk factors need to be defined. Cho, S.Y. et al. showed that in patients with hematologic malignancies, *Stenotrophomonas maltophilia* blood stream infections are associated with a significant mortality rate due to severe neutropenia [18]. The clinical details of 30 hemorrhagic pneumonia cases caused by *Stenotrophomonas maltophilia* infection were compiled in a previous review. The authors found that the most important risk factor for hemorrhagic pneumonia caused by *Stenotrophomonas maltophilia* was severe neutropenia (<100/mm^3^) [29]. In the study of Zhu et al., twenty-four out of twenty-seven (80.9%) patients with *Stenotrophomonas maltophilia* bacteremia with hemorrhagic pneumonia had neutropenia (<500/mm^3^) [38]. In the study of Kim, S.H. et al., hemorrhagic pneumonia was significantly related to persistent neutropenia [32]. This may explain why patients with hematologic malignancy are the only ones who ever suffer *Stenotrophomonas maltophilia*-induced hemorrhagic pneumonia. Susceptibility to *Stenotrophomonas maltophilia* bacteremia and progression to hemorrhagic pneumonia may be influenced by neutropenia caused by the hematologic condition or therapy for the hematologic malignancy. Neutropenia was found to be a risk factor for hemorrhagic pneumonia in the current investigation. Patients who were suffering from *Stenotrophomonas maltophilia* hemorrhagic pneumonia had their neutropenia recovery while receiving therapy, suggesting that neutropenia recovery was crucial to the improved outcomes.

### 4.4. Thrombocytopenia Is a Risk Factor for Hemorrhagic Pneumonia in Stenotrophomonas maltophilia Bacteremia of Hematological Malignancy Patients

Regarding thrombocytopenia, the current study found a significant difference in the percentage of thrombocytopenia between hemorrhagic pneumonia patients and nonhemorrhagic pneumonia patients. Patients may be more susceptible to hemorrhagic pneumonia due to thrombocytopenia and the protease activity level of *Stenotrophomonas maltophilia*. Several studies have revealed that hematologic malignancy and thrombocytopenia are risk factors for hemorrhagic pneumonia in patients with *Stenotrophomonas maltophilia* bacteremia. Imoto, W. et al. showed that four of thirty-five patients with *Stenotrophomonas maltophilia* bacteremia had hemorrhagic pneumonia. Patients with *Stenotrophomonas maltophilia* bacteremia who had hemorrhagic pneumonia had a higher rate of thrombocytopenia than those who did not. The author concluded that thrombocytopenia was a risk factor for *Stenotrophomonas maltophilia*-induced hemorrhagic pneumonia. This might be because those with extremely low platelet counts are more prone to bleeding [36]. In the study of Kim, S.H. et al., according to a clinical review, thrombocytopenia is a key risk factor for hemorrhagic pneumonia [32]. In Zhu’s study, the majority of patients with *Stenotrophomonas maltophilia* bacteremia and hemorrhagic pneumonia also had thrombocytopenia at the same time [38]. Wu, H.G. et al. showed that patients with *Stenotrophomonas maltophilia* respiratory infections often experience thrombocytopenia, which is an independent risk factor for pulmonary hemorrhage and mortality. The author suggested that this group of patients may benefit from a prophylactic platelet transfusion on a regular basis to keep their platelet counts above a certain threshold over unstable periods and may be a potential strategy to serve as a preventative measure against pulmonary hemorrhage [43]. Overall, the current investigation verified that hemorrhagic pneumonia risk factors included thrombocytopenia. More research is required to determine the reason for the decreased platelet counts or function in *Stenotrophomonas maltophilia* bacteremia with hemorrhagic pneumonia. These studies will aid in the treatment of thrombocytopenia and the use of preventative measures.

### 4.5. Prior Antibiotic Therapy Related to Hemorrhagic Pneumonia in Stenotrophomonas maltophilia Bacteremia of Hematological Malignancy Patients

Few studies have investigated the relationship between previous antibiotic usage and mortality risk in hematological malignancy patients with hemorrhagic pneumonia caused by *Stenotrophomonas maltophilia*. Zhu’s study showed that prior tigecycline therapy was an independent risk factor for developing hemorrhagic pneumonia caused by *Stenotrophomonas maltophilia*, which might be attributed to tigecycline side effects [38]. It was previously noted that tigecycline usage might result in coagulopathy, which is typically characterized by a dose-dependent decrease in fibrinogen levels and a prolongation of prothrombin time and activated partial thromboplastin time [44]. This viewpoint was supported by Zhu’s study, which found a greater prevalence of prolonged activated partial thromboplastin time in patients who had previously received tigecycline therapy [38]. Imoto’s study showed that patients who have received quinolone within the past 30 days may be more susceptible to developing hemorrhagic pneumonia caused by *Stenotrophomonas maltophilia*. Quinolones may damage the wall of alveolar microvessels, with the protease released by *Stenotrophomonas maltophilia* causing alveolar microvessel destruction. In addition, thrombocytopenia is common in hematologic malignancy patients, and quinolones are frequently prescribed to this population as a prophylactic antibiotic against bacterial infection. Hemorrhagic pneumonia may be more likely to occur if these processes take place simultaneously [36]. However, this is a hypothesis by the author.

### 4.6. Procalcitonin Is a Risk Factor for Hemorrhagic Pneumonia in Stenotrophomonas maltophilia Bacteremia in Hematological Malignancy Patients

Only Zhu’s study showed that a high procalcitonin level was an independent risk factor for hemorrhagic pneumonia in hematologic patients with *Stenotrophomonas maltophilia* bacteremia [38]. Procalcitonin might mediate inflammation, which can become worse due to the overproduction of early proinflammatory cytokines. The severity of the illness was accompanied by a high level of procalcitonin, which was perhaps linked to severe inflammation that might have damaged the vasculature [45]. This might help to explain why hemorrhagic pneumonia was more likely to proceed in patients with increased procalcitonin.

### 4.7. The Mechanism of Pulmonary Hemorrhage

The precise process of pulmonary hemorrhage is unknown. Numerous virulence factors have been proposed that may be connected to the etiology and clinical presentation of *Stenotrophomonas maltophilia*. The *Stenotrophomonas maltophilia*-secreted protease StmPr1 also breaks down collagen and fibronectin in connective tissue and fibrinogen in blood plasma, killing fibroblasts in vitro. Protease activity in this regard may contribute to the obliteration of alveolar microvessels [46]. In an in vitro study, the protease StmPr1 was shown to cause lung epithelial cells to undergo programmed cell death by detaching themselves from the extracellular matrix and promoting cytokine release [47].

In this review, based on our systematic review of hemorrhagic pneumonia cases in the literature, we describe the clinical characteristics of *Stenotrophomonas maltophilia* bacteremia with hemorrhagic pneumonia. We describe the risk factors and therapeutic approach in the treatment of hemorrhagic pneumonia in patients with hematologic malignancies. The overall survival of hematological patients with *Stenotrophomonas maltophilia* bacteremia may be improved by using a risk stratification strategy, prompt administration of a combination therapy with effective antibiotics, respiratory monitoring, rapid neutrophil recovery, supportive platelet transfusion, and better clinical management of primary hematologic diseases.

## 5. Limitation

The present study has several limitations. First, the number of patients was small. It was difficult to gather enough patients due to the exceedingly low prevalence of *Stenotrophomonas maltophilia* bacteremia with hemorrhagic pneumonia. Second, hemorrhagic pneumonia caused by *Stenotrophomonas maltophilia* is not consistently defined. In the majority of studies, hemorrhagic pneumonia was defined by a patient’s clinical symptoms. Third, *Stenotrophomonas maltophilia* hemorrhagic pneumonia mortality can be decreased by using an adequate combination of antibiotics. The relationships between hemorrhagic pneumonia, antibiotic use, and associated antimicrobial susceptibility, which is important information related to adequate combination antimicrobial therapy, were not examined in the current review.

## 6. Conclusions

The most common underlying hematologic malignancy of hemorrhagic pneumonia patients caused by *Stenotrophomonas maltophilia* bacteremia was acute leukemia. The 7-day mortality rate was 85.9%, and the 30-day mortality rate was 90.1%. *Stenotrophomonas maltophilia* bacteremia with hemorrhagic pneumonia in patients with hematologic malignancies is a situation with rapid development and high mortality. Neutropenia (95%) and thrombocytopenia (86.7%) were frequently observed in hemorrhagic pneumonia patients. Neutropenia and thrombocytopenia were risk factors for hemorrhagic pneumonia in patients with hematologic malignancies with *Stenotrophomonas maltophilia* bacteremia; thus, these patients should be managed with caution.

## 7. Future Directions

In the current review, hemorrhagic pneumonia-related mortality was disproportionately high in patients with hematologic malignancy. Recent literature only has a few cases that were effectively handled. However, there is still no effective therapy that can prevent life-threatening hemorrhagic pneumonia. Hemorrhagic pneumonia is a potentially fatal condition; thus, more research is needed to determine the best ways to prevent it in these patients.

## Figures and Tables

**Table 1:** Characteristics of the included 91 hemorrhagic pneumonia patients.

Author/Year	Region	Mean Age	Underlying Malignancy (No)	Total Patients	Dead Patients
Elsner et al., 1907 [21]	Germany	42.0	AML (2), ALL (1)	3	3
Abbas et al., 2003 [22]	Saudi Arabia	4.9	ALL (3)	3	3
Rousseau et al., 2004 [23]	France	63.0	AML (1)	1	1
Ortin et al., 2007 [24]	Spain	63.0	MDS (1)	1	1
Marinella et al., 2008 [25]	USA	64.0	AML (1)	1	1
Takahashi et al., 2011 [26]	Japan	57.0	Non-Hodgkin lymphoma (1)	1	1
Araoka et al., 2022 [27]	Japan	53.8	AML (7), MDS (2), Non-Hodgkin lymphoma (1)	10	10
Tada et al., 2012 [28]	Japan	42.0	AML (6), Myelofibrosis (1)	7	7
Mori et al., 2014 [29]	Japan	51.5	AML (3), CML (1)	4	4
Gutierrez et al., 2016 [30]	USA	59.0	AML (1)	1	1
Katayama et al., 2017 [31]	Japan	44.0	MDS (1)	1	1
Kim et al., 2018 [32]	Korea	58.9	AML (15), CML (1), Non-Hodgkin lymphoma (3), NK/T-cell leukemia (1)	20	19
Bahatia et al., 2018 [33]	USA	52.0	ALL (1)	1	1
Penagos et al., 2019 [34]	Columbia	21.5	AML (1), ALL (1)	2	0
Andrei et al., 2020 [35]	Romania	61.0	ALL (1)	1	0
Imoto et al., 2020 [36]	Japan	57.5	AML (3), ALL (1)	4	4
Saito et al., 2020 [37]	Japan	46.0	AML (1)	1	0
Zhu et al., 2021 [38]	China	56.0 *	AML (15), ALL (7), MDS (1), Non-Hodgkin lymphoma (3), Hemophagocytic syndrome (1)	27	23
Sarkar et al., 2023 [39]	USA	14.5	AML (2)	2	2

* median age. Foot notes: USA: United States of America; No: number; AML: Acute myeloid leukemia; ALL: Acute lymphoblastic leukemia; CML: Chronic myelogenous leukemia; MDS: Myelodysplastic syndrome.

**Table 2:** Clinical characteristics of hemorrhagic pneumonia related to *Stenotrophomonas maltophoilia* in reported cases (No = 91).

Variables	Hemorrhagic Pneumonia (No) (%)
Age (mean ± SD)	45.19 ± 17.74
Male	54 (59.3%)
Female	37 (40.7%)
Bacteremia	86/91 (94.5%)
Neutropenia	87/91 (95.6%)
Thrombocytopenia	59/68 (86.8%)
7-day mortality	55/64 (85.9%)
30-day mortality	82/91 (90.1%)
**Hematologic Malignancy**	
Acute lymphoblastic leukemia	15 (16.5%)
Acute myeloid leukemia	58 (63.7%)
Chronic myelogenous leukemia	2 (2.3%)
Myelodysplastic syndrome	5 (5.5%)
Non-Hodgkin’s lymphoma	8 (8.8%)
Others	3 (3.2%) *

*: included one case of hemophagocytic syndrome, one case of aggressive NK/T-cell leukemia and one case myelofibrosis. Foot notes: No: number; SD: standard deviation.

## Data Availability

The datasets generated during and/or analyzed during the current study are not publicly available but are available from the corresponding author on reasonable request.
